# Iron(II) supramolecular helicates interfere with the HIV-1 Tat–TAR RNA interaction critical for viral replication

**DOI:** 10.1038/srep29674

**Published:** 2016-07-12

**Authors:** Jaroslav Malina, Michael J. Hannon, Viktor Brabec

**Affiliations:** 1 Institute of Biophysics, Academy of Sciences of the Czech Republic, v.v.i., Kralovopolska 135, CZ-61265 Brno, Czech Republic; 2 School of Chemistry, University of Birmingham, Edgbaston, Birmingham B152TT, United Kingdom; 3 Department of Biophysics, Faculty of Science, Palacky University in Olomouc, Slechtitelu 27, CZ-78371 Olomouc, Czech Republic

## Abstract

The interaction between the HIV-1 transactivator protein Tat and TAR (transactivation responsive region) RNA, plays a critical role in HIV-1 transcription. Iron(II) supramolecular helicates were evaluated for their *in vitro* activity to inhibit Tat–TAR RNA interaction using UV melting studies, electrophoretic mobility shift assay, and RNase A footprinting. The results demonstrate that iron(II) supramolecular helicates inhibit Tat-TAR interaction at nanomolar concentrations by binding to TAR RNA. These studies provide a new insight into the biological potential of metallosupramolecular helicates.

The trans activation response region (TAR) of RNA (Fig. 1b) represents an attractive target for the inhibition of human imunodeficiency virus type 1 (HIV-1) replication by small molecules. The binding of the viral trans activator protein (Tat) to the TAR RNA is an essential step in the HIV-1 replication cycle. Therefore, the blockage of the Tat-TAR interaction is a potential route for AIDS chemotherapy. Compounds that bind to TAR RNA, and prevent binding by Tat, could disrupt processive transcription and thereby inhibit viral growth^1^.

Tat binds to TAR RNA at the three-nucleotide pyrimidine bulge and interacts with two base pairs above and below the bulge in the major groove of the TAR RNA molecule^2^,^3^. Upon binding of Tat, TAR RNA undergoes a distinct conformational change characterized by a significant compression of the protein binding pocket at the pyrimidine bulge^4^,^5^. Numerous small molecules and peptides that bind three-nucleotide bulges have been synthesized and tested in the past^6^,^7^,^8^,^9^,^10^, but many of these molecules did not have sufficient potency to progress into pharmaceutical development^11^.

We have shown that dinuclear iron (II) metallosupramolecular triple helicates [Fe_2_L_3_]Cl_4_ (L = C_25_H_20_N_4_, Fig. 1a) can specifically recognize various unusual DNA or RNA structures, such as Y-shaped three-way junctions^12^,^13^,^14^, three-way junctions containing unpaired nucleotides, the so-called T-shaped three-way junctions^15^ and others also studied binding to human telomeric G-quadruplex DNA^16^. Interestingly, these helicates, similarly as a flexibly-linked dinuclear ruthenium(II) complex^17^,^18^, can also bind nucleic acids bulges containing two or more unpaired nucleotides^19^. The three-nucleotide pyrimidine bulge in TAR RNA represents a binding site for the recruitment of the viral transactivator protein Tat. For this reason we were intrigued to see if the cylindrical helicates would bind to the bulge of TAR RNA and in this way could block the Tat-TAR RNA interaction.

Here, we report on the interactions of *M*- and *P*-enantiomers of [Fe_2_L_3_]Cl_4_ with the TAR RNA by using thermal denaturation, electrophoretic mobility shift assay, and RNase A footprinting. In addition, we used the ADP-1 polypeptide (Fig. 1c) that carries the minimal RNA recognition region of the Tat protein and closely mimics Tat binding specificity^3^,^4^ to investigate whether the helicates can inhibit formation of the Tat-TAR RNA complex.

## Results and Discussion

### Binding of the helicates to the HIV-1 TAR RNA

#### UV melting studies

**Figure 1:**
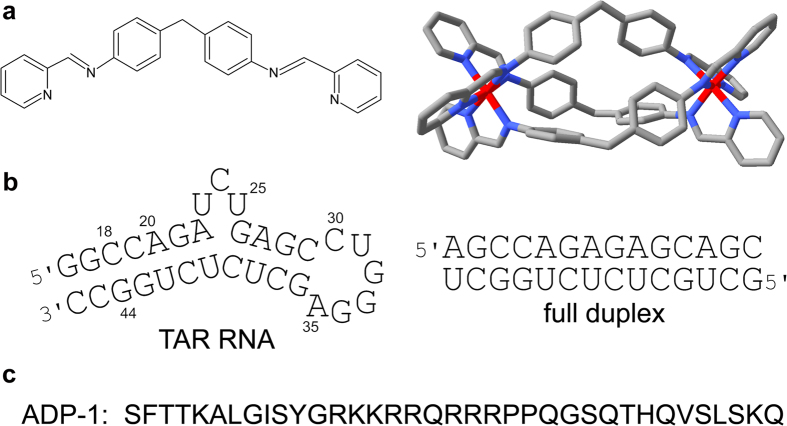
Structures of helicates, RNA nucleotide sequences and amino acid sequence of the ADP-1 polypeptide. (**a**) Structures of the ligand L and the tetracataionic triple helicate *M*-[Fe_2_L_3_]^4+^ formed from that ligand [adapted from Protein Data Bank (PDB) file 2ET0]. (**b**) Sequences of the TAR RNA, containing residues 18–44 of HIV-1 mRNA and two additional G·C base pairs, and the fully matched RNA duplex. (**c**) The sequence of the ADP-1 polypeptide carrying the minimal RNA recognition region of the Tat protein (residues 37–72) and closely mimicking Tat binding specificity.

The ability of *M*- and *P*-[Fe_2_L_3_]Cl_4_ to alter the melting temperature (*T*_m_) of TAR RNA reflects to some extend the strength of binding. The melting curves of TAR RNA and fully matched RNA duplex (Fig. 1b) in the absence and presence of the helicates are shown in Supplementary Figs S1and S2 online. The Δ*T*_m_ (*T*_m_ of the helicate-RNA complex - *T*_m_ of the free RNA) values are presented in Table 1.

The melting temperatures of the TAR RNA and fully matched RNA duplex in the absence of the helicates were 67.4 °C and 65.7 °C, respectively. The presence of *M*- and *P*-[Fe_2_L_3_]Cl_4_ increased the *T*_m_ of the TAR RNA by 12.1 °C and 11.2 °C at 1:1 helicate:RNA ratio, respectively, and by 13.3 °C and 12.9 °C at 2:1 helicate:RNA ratio, respectively. Doubling the helicate:RNA ratio from 1:1 to 2:1 had little effect (<2 °C) on the thermal stability of the TAR RNA, which indicates that there is a single dominant binding site for the helicates on the TAR RNA. The *T*_m_ values of the fully matched RNA duplex were affected only negligibly by the presence of the helicates.

Binding constants (*K*) of the helicates to TAR RNA, calculated from ethidium bromide displacement experiments (Supplementary Fig. S3) revealed that both *M-*[Fe_2_L_3_]Cl_4_ and *P*-[Fe_2_L_3_]Cl_4_ helicates exhibit similar binding affinities for the TAR RNA (the values of *K* were 190 ± 10 × 10^6^ M^−1^ and 222 ± 13 × 10^6^ M^−1^ for the *M*- and *P*-enantiomer, respectively).

#### Electrophoretic mobility shift assay

**Figure 2:**
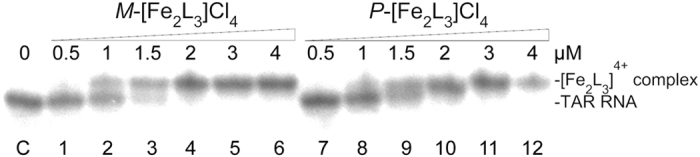
Binding of the helicates to TAR RNA. Autoradiogram of the gel run at 5 °C showing binding of the helicates to TAR RNA (2 μM). Lane C: TAR-RNA in the absence of the helicates. Lanes 1–6: TAR RNA mixed with *M*-[Fe_2_L_3_]Cl_4_ at 0.25:1, 0.5:1, 0.75:1, 1:1, 1.5:1 and 2:1 (helicate:TAR RNA) ratios, respectively. Lanes 7–12: TAR RNA mixed with *P*-[Fe_2_L_3_]Cl_4_ at 0.25:1, 0.5:1, 0.75:1, 1:1, 1.5:1 and 2:1 (helicate:TAR RNA) ratios, respectively.

An electrophoretic mobility shift assay (EMSA) was used to explore the stability of the helicate:TAR RNA complex. The autoradiogram of the electrophoresis gel run at 5 °C (Fig. 2) shows the interaction of the TAR RNA with increasing concentrations of *M*- and *P*-[Fe_2_L_3_]Cl_4_. It can be seen that a new, more slowly migrating band indicating the formation of the helicate:TAR RNA complex appears in the gel in the presence of the helicates.

Inspection of the gel in Fig. 2 also shows that when the helicate:TAR RNA ratio exceeds 1:1 no additional bands indicating the formation of the TAR RNA complex with two molecules of [Fe_2_L_3_]^4+^ appear in the gel.

It supports the presence of only one major binding site for the helicates on the TAR RNA. We performed the same experiment under the same conditions using the fully matched RNA duplex but no new bands corresponding to the formation of the helicate:RNA complex were observed in the gel (see Supplementary Fig. S4).

#### RNase A footprinting

**Figure 3:**
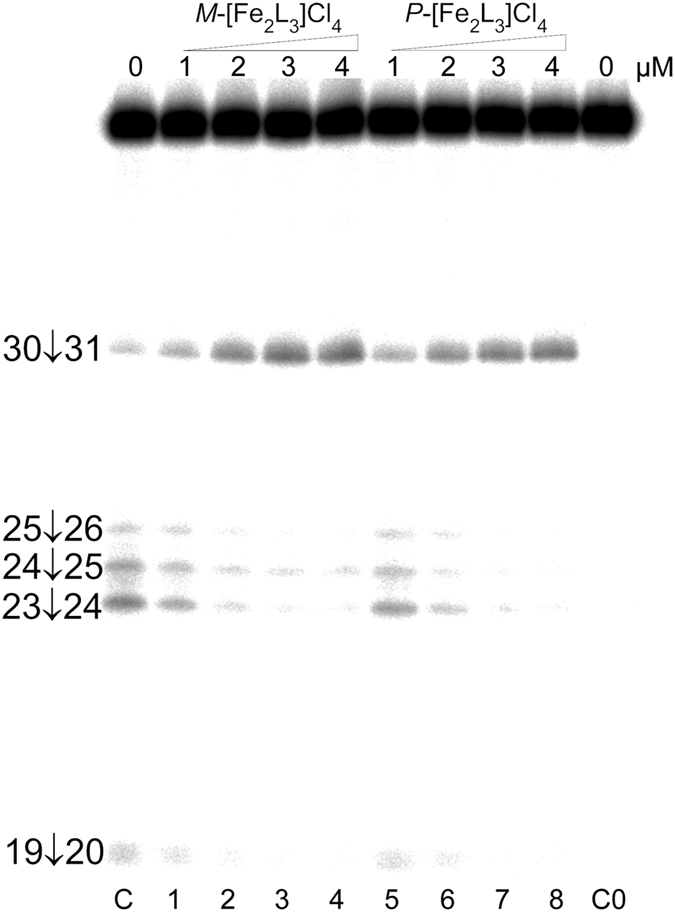
RNase A cleavage of TAR RNA in the presence of helicates. RNase A cleavage of 5′-^32^P end-labeled TAR RNA (2 μM) in the presence of increasing concentrations of *M*- and *P*-[Fe_2_L_3_]Cl_4_. Lane C; TAR RNA cleaved by RNase A in the absence of helicates. Lanes 1–4; TAR RNA mixed with *M*-[Fe_2_L_3_]Cl_4_ at 0.5:1, 1:1, 1.5:1, and 2:1 (helicate:TAR RNA) ratios, respectively, cleaved by RNase A. Lanes 5–8; TAR RNA mixed with *P*-[Fe_2_L_3_]Cl_4_ at 0.5:1, 1:1, 1.5:1, and 2:1 (helicate:TAR RNA) ratios, respectively, cleaved by RNase A. Lane C0; TAR RNA in the absence of helicates and RNase A. Phosphodiester bonds cleaved by RNase A are indicated on the left side of the gel.

In order to identify potential binding sites of *M*- and *P*-[Fe_2_L_3_]Cl_4_ on the TAR RNA, we probed the helicate:TAR RNA complexes with RNase A. Since this enzyme prefers cleavage of single-stranded to double-stranded regions of the RNA, it is particularly well suited for investigating drug binding to the single stranded parts, including the bulge and loop regions. The autoradiogram of the RNA cleavage-inhibition patterns for the TAR RNA is shown in Fig. 3. The strong cleavage at positions 23–26 corresponding to the 5′-UCU bulge is markedly reduced in the presence of both enantiomers of [Fe_2_L_3_]Cl_4_. In contrast, the cutting is increased between nucleotides C30 and U31 located in the loop. It is likely that the base protection effects induced by the helicates result from the direct interaction with the helicates, but it cannot be excluded that the protection arises from helicate-induced structural changes. In any case, the results of the RNase A footprinting suggest that the *M*- and *P*-[Fe_2_L_3_]Cl_4_ preferentially bind to the TAR RNA bulge or in its close proximity.

#### Inhibition of the HIV-1 Tat-TAR interaction

**Figure 4:**
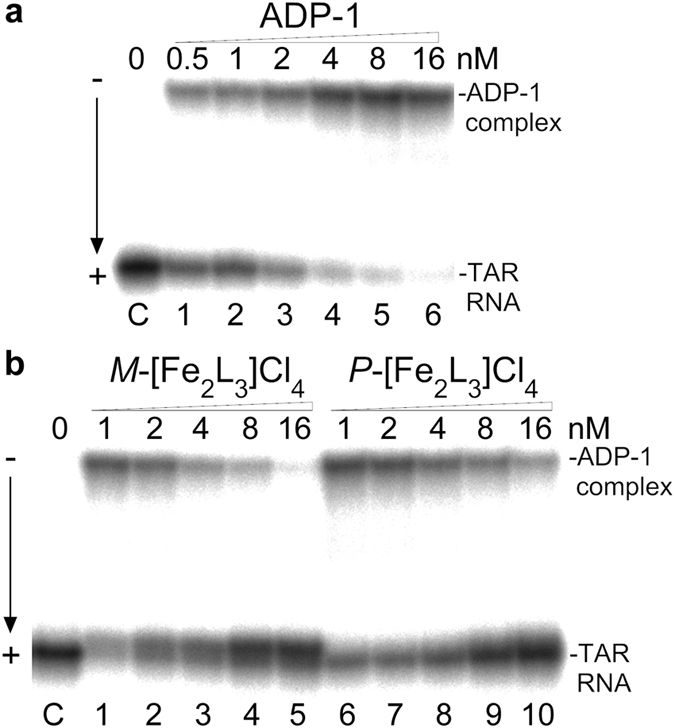
Formation of the complex between the ADP-1 peptide and TAR RNA and inhibition of the formation of the complex by helicates. (**a**) Binding of the ADP-1 peptide to the TAR RNA (2 nM). Lane C; TAR RNA in the absence of the ADP-1. Lanes 1–6; TAR RNA in the presence of 0.5, 1, 2, 4, 8, and 16 nM ADP-1, respectively. (**b**) Inhibition of the complex between the ADP-1 peptide (4 nM) and TAR RNA (2 nM) by *M*- and *P*-[Fe_2_L_3_]Cl_4_. Lane C; TAR RNA in the absence of the ADP-1 and helicates. Lanes 1–5; TAR RNA mixed with the ADP-1 and increasing concentrations (1, 2, 4, 8, and 16 nM, respectively) of *M*-[Fe_2_L_3_]Cl_4_. Lanes 6–10; TAR RNA mixed with the ADP-1 and increasing concentrations (1, 2, 4, 8, and 16 nM, respectively) of *P*-[Fe_2_L_3_]Cl_4_.

The EMSA was also employed to determine if the binding of the helicates to the TAR RNA can inhibit the Tat-TAR interaction. In these experiments, we used the ADP-1 polypeptide (see Fig. 1c for its sequence) that has been previously demonstrated^4^ to carry the minimal RNA recognition region of the HIV-1 Tat protein and closely mimic Tat binding specificity. The autoradiogram in Fig. 4a presents binding of the ADP-1 to the TAR RNA in the absence of the helicates. The autoradiogram in Fig. 4b shows how the formation of the ADP-1-TAR RNA complex is inhibited in the presence of *M*- and *P*-[Fe_2_L_3_]Cl_4_. It can be seen that the ADP-1-TAR RNA interaction is almost completely inhibited in the presence of both enantiomers at the concentration of 16 nM. It might be possible that the helicates disrupt formation of the ADP-1-TAR complex by binding to the peptide rather than to the TAR-RNA. In order to probe this eventuality, we recorded CD spectra of *M*-[Fe_2_L_3_]Cl_4_ at constant helicate concentration (14 μM) and increasing ADP-1 concentrations (see Supplementary Fig. S5). No changes in the CD spectra were noticed with increasing concentrations of ADP-1, which is consistent with the view that the helicates did not bind significantly to ADP-1 peptide under conditions of our experiments.

In conclusion, our results show that both *M*- and *P*-[Fe_2_L_3_]Cl_4_ bind with high affinity to the TAR RNA and discriminate this three-base bulge containing RNA against fully matched RNA duplex. It is reasonable to expect that the substantial contribution to the binding affinity of the helicates to TAR RNA comes from their strong binding affinity to the structural motif, such as the triangular prismatic pocket formed by the unpaired bulge bases, to accommodate the helicate molecule^19^. RNase A footprinting indicates that helicates bind directly to the 5′-UCU bulge or in its close proximity. Furthermore, our results demonstrate that nanomolar concentrations of both enantiomers disrupt formation of the Tat-TAR complex in a purified protein-RNA system. Thus, iron(II) supramolecular helicates inhibit the HIV-1 Tat-TAR interaction at notably lower concentrations than many inhibitors of Tat-TAR binding so far tested^11^,^20^,^21^,^22^,^23^. Iron(II) supramolecular helicates provide reasonable templates for further developing TAR ligands with improved efficiency and selectivity capable of inhibiting the functions of their RNA target. Further investigation of these helicates and their new derivatives more selective for TAR RNA in HIV-1-infected cellular environments is required for better understanding of their efficacies in HIV-1 therapy which can lead to the discovery of novel and highly potent antivirals.

## Methods

### Chemicals

The iron(II) helicates [Fe_2_L_3_]Cl_4_ (L = C_25_H_20_N_4_; Fig. 1a) were synthesised as previously described^24^,^25^,^26^. The synthetic oligoribonucleotides used in this work were purchased from VBC-Biotech (Vienna, Austria). The ADP-1 polypeptide (Fig. 1c) was purchased from Schafer-N (Copenhagen, Denmark) and was >95% pure. T4 polynucleotide kinase was purchased from New England Biolabs (Beverly, MA). [γ-^32^P]-ATP was from MP Biomedicals, LLC (Irvine, CA). Acrylamide and bis(acrylamide) were from Merck KgaA (Darmstadt, Germany). RNase A was from Roche (Mannheim, Germany).

### UV melting experiments

The stability of DNA bulges in the presence of the helicates was monitored by measuring the absorbance at 260 nm (1 nm bandwidth, average time: 10 s, heating rate 0.4 °C/min) as a function of temperature. The experiment was run simultaneously on six masked 1 cm pathlength microcuvettes of 0.2 mL volume using a Peltier controlled 6-sample cell-changer in a Varian Cary 4000 UV/vis spectrophotometer. Melting temperature (*T*_m_) was calculated within the thermal heating program by applying a first derivative calculation. The *T*_m_ values could be determined with an accuracy of ±0.5 °C. Each DNA melting experiment was carried out at least three times and the *T*_m_ values represent the mean. The concentration of oligoribonucleotides was 3 μM per strand. The buffer conditions were sodium phosphate buffer (10 mM, pH 7.0) and 0.5 mM EDTA.

### Electrophoretic mobility shift assays

RNA was 5′-end labeled using T4 polynucleotide kinase and [γ-^32^P]ATP and then it was purified by denaturing polyacrylamide gel electrophoresis (PAGE). The RNA concentration was determined by the UV absorbance at 260 nm. RNA was annealed by heating at 90 °C and slow cooling to room temperature in sterile water. RNA, helicates, and the ADP-1 peptide were mixed in a buffer (5 μL) containing Tris-HCl (50 mM, pH 8.0), KCl (50 mM), DTT (100 mM), and Triton X-100 (0.05%) and incubated for 10 min on ice. The samples were analysed by electrophoresis on 15% polyacrylamide (PAA) gels in 0.5 × TB buffer and electrophoresed at 5 W and 5 °C. Gels were exposed to a phosphor imaging plate and scanned with a FUJIFILM BAS-2500 bio-imaging analyzer.

### RNase A footprinting

TAR RNA was 5′-end labeled using T4 polynucleotide kinase and [γ-^32^P]ATP and then purified by denaturing PAGE. TAR RNA was annealed by heating at 90 °C and slow cooling to room temperature in sterile water. 5 μL solutions containing 2 × 10^−6^ M TAR RNA in 10 mM Tris-HCl (pH 7.4) and various concentrations of the helicates were incubated for 10 min at 25 °C. Cleavage was initiated by the addition of 1 μL of RNase A diluted in the precedent experiment to the concentration (5 × 10^−7^ unit) that was sufficient to achieve partial cleavage of the TAR RNA. Samples were allowed to react for 5 min at room temperature before quenching with 5 μL of 2× concentrated formamide loading buffer followed by incubation at 90 °C for 3 min. 2 μL of the mixture containing RNA cleavage products were then withdrawn and resolved by PAGE under denaturing conditions (24%/8 M urea PAA gel). Gels were exposed to a phosphor imaging plate and scanned with a FUJIFILM BAS-2500 bio-imaging analyzer.

## Additional Information

**How to cite this article**: Malina, J. *et al*. Iron(II) supramolecular helicates interfere with the HIV-1 Tat-TAR RNA interaction critical for viral replication. *Sci. Rep.* **6**, 29674; doi: 10.1038/srep29674 (2016).

## Supplementary Material

Supplementary Information

## Figures and Tables

**Table 1:** Thermal stability of the fully matched RNA duplex and the TAR RNA in the presence of *M*- and *P*-[Fe_2_L_3_]Cl_4_.

Compound	Δ*T*_m_ (°C) at 1:1^a^	Δ*T*_m_ (°C) at 2:1^b^
TAR RNA (*T*_m_ = 67.4 °C)		
* M-*[Fe_2_L_3_]Cl_4_	12.1	13.3
* P*-[Fe_2_L_3_]Cl_4_	11.2	12.8
Fully matched RNA duplex (*T*_m_ = 65.7 °C)		
* M-*[Fe_2_L_3_]Cl_4_	−0.5	−1.0
* P*-[Fe_2_L_3_]Cl_4_	−0.1	0.5

^a^Helicate:duplex was 1:1.

^b^Helicate:duplex was 2:1.
