# Supplementary material for: Iron(II) supramolecular helicates interfere with the HIV-1 Tat–TAR RNA interaction critical for viral replication
Source: Sci Rep. 2016 Jul 12;6:29674. doi: 10.1038/srep29674 (PMC4940744; doi:10.1038/srep29674)
Supplement: Supplementary Information [file srep29674-s1.pdf]

Supplementary Material

Iron(II) supramolecular helicates interfere with the HIV-1 Tat−TAR RNA interaction critical for viral replication

Jaroslav Malina*[1], Michael J. Hannon[2] & Viktor Brabec[1,3]

[1] Institute of Biophysics, Academy of Sciences of the Czech Republic, v.v.i., Kralovopolska 135, CZ-61265 Brno, Czech Republic

[2] School of Chemistry, University of Birmingham, Edgbaston, Birmingham B152TT, United Kingdom

[3] Department of Biophysics, Faculty of Science, Palacky University in Olomouc, Slechtitelu 27, CZ-78371 Olomouc, Czech Republic

Table of Contents

RESULTS S2

Melting of the TAR RNA and fully matched RNA duplex in the presence of helicates (Figure S1) S2

The first derivatives of the melting curves for TAR RNA in the presence of M- and P-[Fe_2L_3]Cl_4 (Figure S2) S3

Titration of M- and P-[Fe_2L_3]Cl_4 against TAR RNA with ethidium bromide (Figure S3) S3

Absence of binding of the helicates to fully matched RNA duplex (Figure S4) S4

CD spectra for mixtures of M-[Fe_2L_3]Cl_4 and ADP-1 peptide (Figure S5) S4

RESULTS

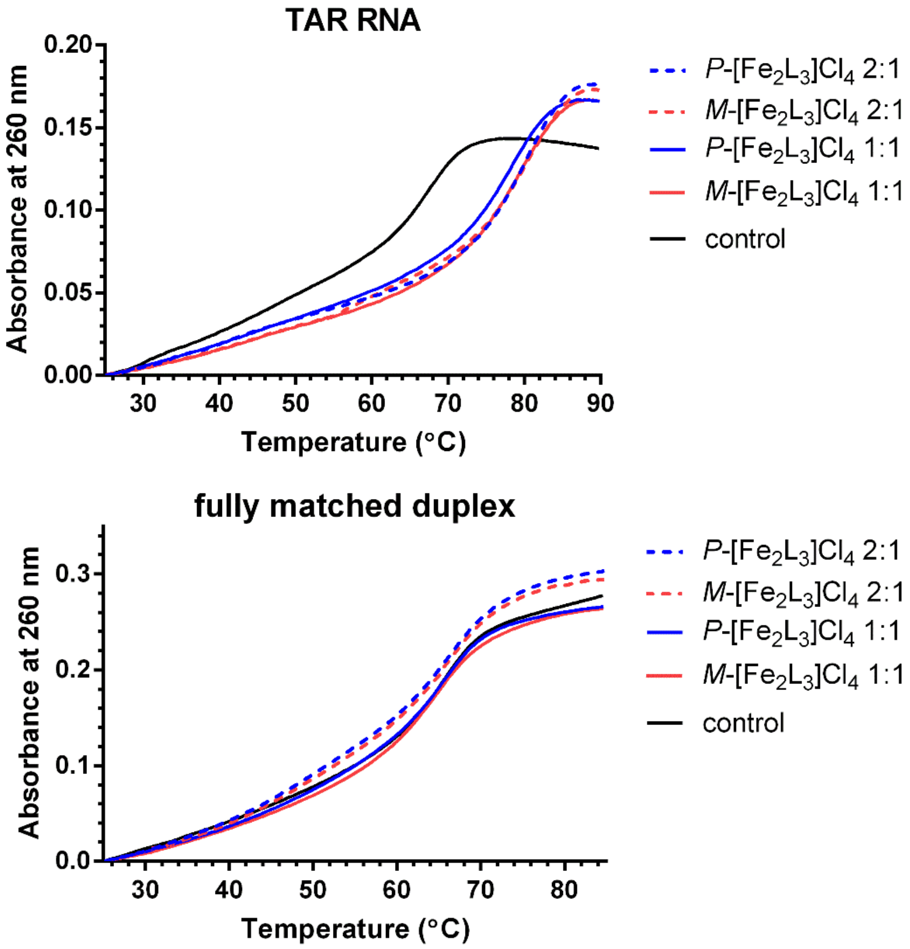

Supplementary Figure S1: Melting of the TAR RNA and fully matched RNA duplex in the presence of helicates

Melting curves for the TAR RNA (3 μM) and fully matched RNA duplex (3 μM) in the presence of *M*- and *P*-$[Fe_2L_3]Cl_4$ at 1:1 and 2:1 helicate:RNA ratios.

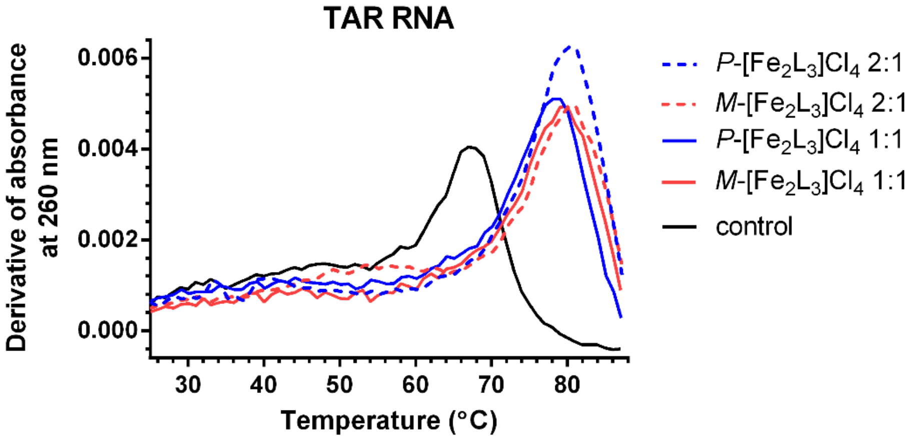

Supplementary Figure S2: The first derivatives of the melting curves for TAR RNA in the presence of *M*- and *P*-[Fe_2L_3]Cl_4

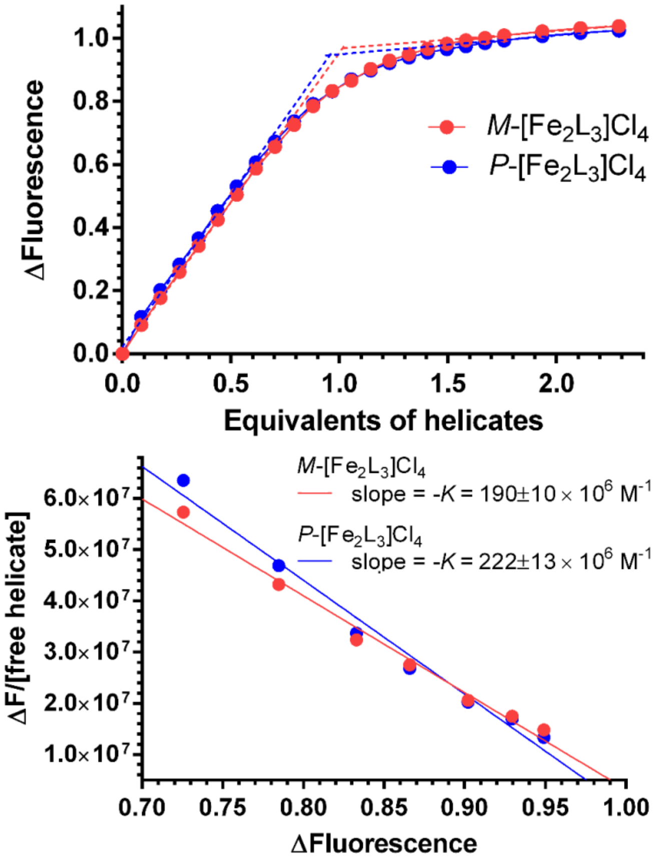

Supplementary Figure S3: Titration of *M*- and *P*-[Fe_2L_3]Cl_4 against TAR RNA at 0.2 µM with ethidium bromide and the Scatchard plot for determining K_{app}

A 3 ml quartz cuvette was loaded with 2.5 mL of 10 mM sodium phosphate buffer (pH 7.0) and 1.2 µM ethidium bromide. The fluorescence was measured on Varian Cary Eclipse spectrofluorometer (ex. 545 nm, em. 595 nm) and normalized to 0% fluorescence. The TAR RNA was added (0.2 µM final concentration) and the resulting fluorescence was normalized to 100%. Titrations were performed by adding aliquots of helicates and measuring the decrease of fluorescence after a 5 min equilibration time.

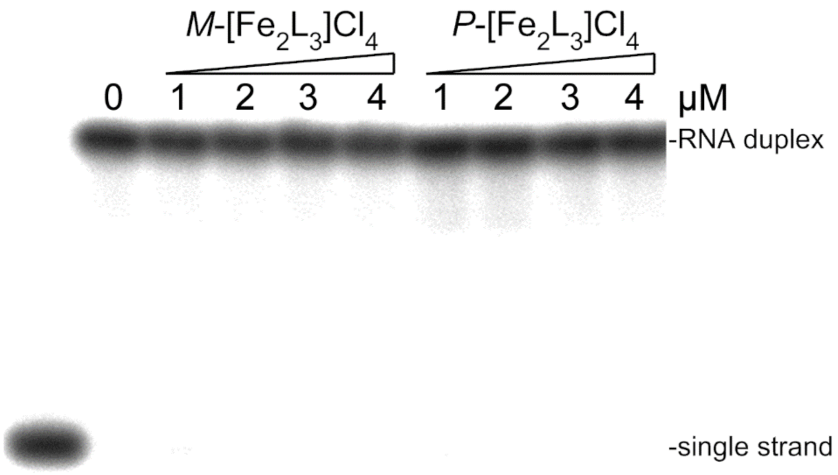

Supplementary Figure S4: Absence of binding of the helicates to fully matched RNA duplex

Autoradiogram of the gel run at 5 ˚C showing absence of binding of the helicates to fully matched RNA duplex (2 μM). Lane ss: top strand in the absence of the helicates. Lane C: RNA duplex in the absence of the helicates. Lanes 1-4: RNA duplex mixed with *M*-[Fe_2L_3]Cl_4 at 0.5:1, 1:1, 1.5:1 and 2:1 helicate:RNA duplex ratios, respectively. Lanes 5-8: RNA duplex mixed with *P*-[Fe_2L_3]Cl_4 at 0.5:1, 1:1, 1.5:1 and 2:1 helicate:RNA duplex ratios, respectively.

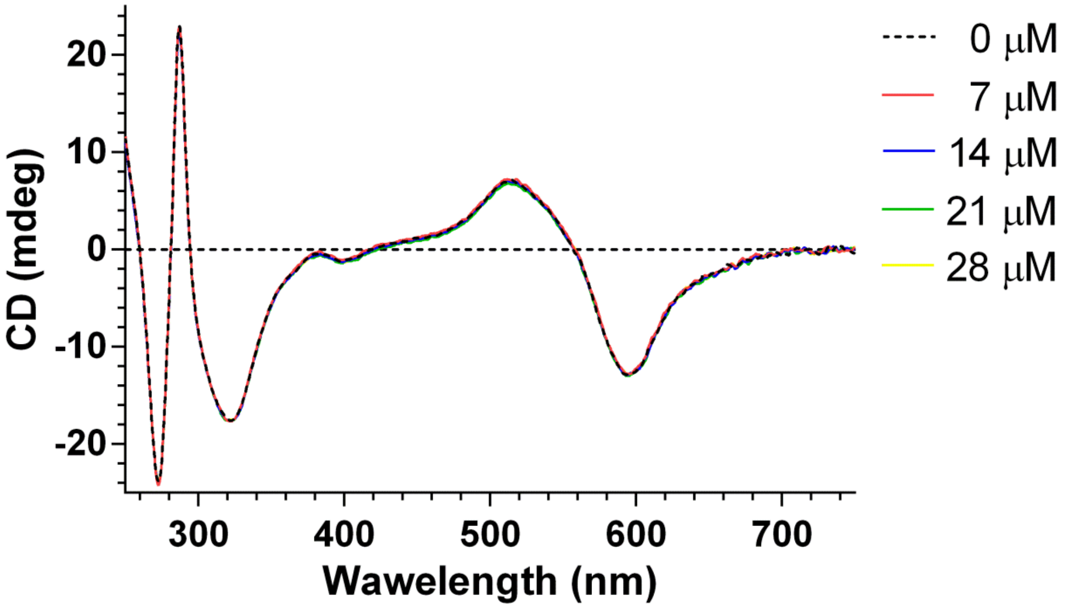

Supplementary Figure S5: CD spectra for the mixture of for *M*-[Fe_2L_3]Cl_4 and ADP-1 peptide

CD titration series for *M*-[Fe_2L_3]Cl_4 at constant helicate concentration (14 μM) and increasing ADP-1 concentrations (indicated in legend). The buffer composition was 50 mM Tris-HCl (pH 8.0) and 50 mM KCl. Cuvette path length was 1.0 cm.
